# Supplementary material for: The Lamin B receptor is essential for cholesterol synthesis and perturbed by disease-causing mutations
Source: eLife. 2016 Jun 23;5:e16011. doi: 10.7554/eLife.16011 (PMC4951196; doi:10.7554/eLife.16011)
Supplement: Figure 3—source data 1. — The TM7SF2 cDNA sequence cloned from HeLa cells (top strand) was aligned with the coding sequence of the published human TM7SF2 transcript isoform1 (NM_003273) (lower strand). DOI: http://dx.doi.org/10.7554/eLife.16011.010 [file elife-16011-fig3-data1.pdf]

DNA Strider 1.4f6 ### Wednesday, June 15, 2016 10:30:59 AM (US Letter @ 80%)

HeLaTM7SF2.xdna x NCBI_transcript1.xdna [163 to 1419] => DNA Parallel

```
DNA  sequence      1401  bp   GTCTGCGTTCCG ... TACATCTACTGA   linear

DNA  sequence      1614  bp   GGGACGAGAGCG ... AAAAAAAAAAAA   linear


   Method:           Blocks (Martinez)
   Layout:           Standard
   Mismatch penalty: Smaller (1)
   Gap penalty:      Medium  (2)
   Translation:      Bottom only

                 •         20        •         40        •         60        •         80
      1 GTCTGCGTTCCGTGTCCAGGCAGGTGCAGGCGCCGCGGGGCCGGATCCTCCGCGCGGCCGAGTCCATCTCCTGGGAAATG 80

        ================================================================================


                 •        100        •        120        •        140        •        160
     81 GGGCGGACAGTGTTTCCTTGACTGACTATTGTGAGCGCCCTCTCTCTCCGGCGGAGCGGAGACCATGGCCCCCACTCAGG 160
                                                                        ||||||||||||||||
    163 ================================================================ATGGCCCCCACTCAGG 178
                                                                         M  A  P  T  Q  G
                                                                                  •
                 •        180        •        200        •        220        •        240
    161 GCCCCCGGGCCCCGCTGGAATTCGGAGGGCCCCTGGGCGCCGCGGCTCTGCTACTGCTGCTGCCCGCCACCATGTTCCAC 240
        ||||||||||||||||||||||||||||||||||||||||||||||||||||||||||||||||||||||||||||||||
    179 GCCCCCGGGCCCCGCTGGAATTCGGAGGGCCCCTGGGCGCCGCGGCTCTGCTACTGCTGCTGCCCGCCACCATGTTCCAC 258
          P  R  A  P  L  E  F  G  G  P  L  G  A  A  A  L  L  L  L  L  P  A  T  M  F  H
      180        •        200        •        220        •        240        •
                 •        260        •        280        •        300        •        320
    241 CTGCTCCTGGCGGCCCGTTCGGGCCCCGCGCGCCTGCTGGGTCCACCCGCGTCCCTGCCCGGGCTGGAGGTGCTGTGGAG 320
        ||||||||||||||||||||||||||||||||||||||||||||||||||||||||||||||||||||||||||||||||
    259 CTGCTCCTGGCGGCCCGTTCGGGCCCCGCGCGCCTGCTGGGTCCACCCGCGTCCCTGCCCGGGCTGGAGGTGCTGTGGAG 338
         L  L  L  A  A  R  S  G  P  A  R  L  L  G  P  P  A  S  L  P  G  L  E  V  L  W  S
      260        •        280        •        300        •        320        •
                 •        340        •        360        •        380        •        400
    321 CCCACGGGCGCTGCTGCTGTGGCTCGCCTGGCTCGGCCTGCAGGCGGCGCTCTACCTACTGCCGGCGCGCAAGGTGGCCG 400
        ||||||||||||||||||||||||||||||||||||||||||||||||||||||||||||||||||||||||||||||||
    339 CCCACGGGCGCTGCTGCTGTGGCTCGCCTGGCTCGGCCTGCAGGCGGCGCTCTACCTACTGCCGGCGCGCAAGGTGGCCG 418
          P  R  A  L  L  L  W  L  A  W  L  G  L  Q  A  A  L  Y  L  L  P  A  R  K  V  A  E
      340        •        360        •        380        •        400        •
                 •        420        •        440        •        460        •        480
    401 AGGGGCAGGAATTGAAGGACAAGAGTCGCCTGCGCTATCCTATTAACGGCTTCCAGGCCCTGGTGCTGACAGCCCTGTTG 480
        ||||||||||||||||||||||||||||||||||||||||||||||||||||||||||||||||||||||||||||||||
    419 AGGGGCAGGAATTGAAGGACAAGAGTCGCCTGCGCTATCCTATTAACGGCTTCCAGGCCCTGGTGCTGACAGCCCTGTTG 498
          G  Q  E  L  K  D  K  S  R  L  R  Y  P  I  N  G  F  Q  A  L  V  L  T  A  L  L
      420        •        440        •        460        •        480        •
                 •        500        •        520        •        540        •        560
    481 GTGGGGCTGGGGATGTCAGCGGGGCTGCCTCTGGGGGCGCTCCCGGAAATGCTCCTGCCCTTGGCGTTTGTCGCCACCCT 560
        ||||||||||||||||||||||||||||||||||||||||||||||||||||||||||||||||||||||||||||||||
    499 GTGGGGCTGGGGATGTCAGCGGGGCTGCCTCTGGGGGCGCTCCCGGAAATGCTCCTGCCCTTGGCGTTTGTCGCCACCCT 578
         V  G  L  G  M  S  A  G  L  P  L  G  A  L  P  E  M  L  L  P  L  A  F  V  A  T  L
      500        •        520        •        540        •        560        •
                 •        580        •        600        •        620        •        640
    561 CACCGCTTTCATCTTCAGCCTCTTTCTCTACATGAAGGCGCAGGTAGCCCCAGTTTCGGCCCTGGCACCTGGGGGGAACT 640
        ||||||||||||||||||||||||||||||||||||||||||||||||||||||||||||||||||||||||||||||||
    579 CACCGCTTTCATCTTCAGCCTCTTTCTCTACATGAAGGCGCAGGTAGCCCCAGTTTCGGCCCTGGCACCTGGGGGGAACT 658
          T  A  F  I  F  S  L  F  L  Y  M  K  A  Q  V  A  P  V  S  A  L  A  P  G  G  N  S
      580        •        600        •        620        •        640        •
                 •        660        •        680        •        700        •        720
    641 CAGGCAATCCGATTTACGACTTTTTTCTGGGACGAGAGCTCAACCCTCGTATCTGTTTCTTCGACTTCAAATATTTCTGT 720
        ||||||||||||||||||||||||||||||||||||||||||||||||||||||||||||||||||||||||||||||||
    659 CAGGCAATCCGATTTACGACTTTTTTCTGGGACGAGAGCTCAACCCTCGTATCTGTTTCTTCGACTTCAAATATTTCTGT 738
          G  N  P  I  Y  D  F  F  L  G  R  E  L  N  P  R  I  C  F  F  D  F  K  Y  F  C
      660        •        680        •        700        •        720        •
                 •        740        •        760        •        780        •        800
    721 GAACTGCGACCCGGCCTCATCGGCTGGGTCCTCATCAACCTGGCCCTGTTGATGAAGGAGGCAGAGCTTCGAGGCAGTCC 800
        ||||||||||||||||||||||||||||||||||||||||||||||||||||||||||||||||||||||||||||||||
    739 GAACTGCGACCCGGCCTCATCGGCTGGGTCCTCATCAACCTGGCCCTGTTGATGAAGGAGGCAGAGCTTCGAGGCAGTCC 818
         E  L  R  P  G  L  I  G  W  V  L  I  N  L  A  L  L  M  K  E  A  E  L  R  G  S  P
      740        •        760        •        780        •        800        •
                 •        820        •        840        •        860        •        880
    801 CTCACTGGCCATGTGGCTGGTCAATGGCTTCCAGTTGCTCTACGTGGGTGATGCCCTCTGGCACGAGGAGGCCGTCCTCA 880
        ||||||||||||||||||||||||||||||||||||||||||||||||||||||||||||||||||||||||||||||||
    819 CTCACTGGCCATGTGGCTGGTCAATGGCTTCCAGTTGCTCTACGTGGGTGATGCCCTCTGGCACGAGGAGGCCGTCCTCA 898
          S  L  A  M  W  L  V  N  G  F  Q  L  L  Y  V  G  D  A  L  W  H  E  E  A  V  L  T
      820        •        840        •        860        •        880        •
```

```
                    •        900        •        920        •        940        •        960
     881 CCACCATGGATATCACACATGACGGGTTTGGCTTCATGCTGGCGTTTGGGGACATGGCCTGGGTGCCCTTCACCTACAGC 960
         ||||||||||||||||||||||||||||||||||||||||||||||||||||||||||||||||||||||||||||||||
     899 CCACCATGGATATCACACATGACGGGTTTGGCTTCATGCTGGCGTTTGGGGACATGGCCTGGGTGCCCTTCACCTACAGC 978
           T  M  D  I  T  H  D  G  F  G  F  M  L  A  F  G  D  M  A  W  V  P  F  T  Y  S
      900         •        920        •        940        •        960        •
                    •        980        •       1000        •       1020        •       1040
     961 CTGCAGGCCCAGTTCCTGCTGCACCACCCGCAGCCCCTGGGGTTGCCCATGGCCTCTGTCATCTGCCTCATCAATGCTAC 1040
         ||||||||||||||||||||||||||||||||||||||||||||||||||||||||||||||||||||||||||||||||
     979 CTGCAGGCCCAGTTCCTGCTGCACCACCCGCAGCCCCTGGGGTTGCCCATGGCCTCTGTCATCTGCCTCATCAATGCTAC 1058
          L  Q  A  Q  F  L  L  H  H  P  Q  P  L  G  L  P  M  A  S  V  I  C  L  I  N  A  T
      980         •       1000        •       1020        •       1040        •
                    •       1060        •       1080        •       1100        •       1120
    1041 TGGTTACTACATCTTCCGTGGGGCGAATTCCCAGAAAAACACTTTCCGAAAGAATCCTTCTGACCCCAGAGTGGCTGGGC 1120
         ||||||||||||||||||||||||||||||||||||||||||||||||||||||||||||||||||||||||||||||||
    1059 TGGTTACTACATCTTCCGTGGGGCGAATTCCCAGAAAAACACTTTCCGAAAGAATCCTTCTGACCCCAGAGTGGCTGGGC 1138
           G  Y  Y  I  F  R  G  A  N  S  Q  K  N  T  F  R  K  N  P  S  D  P  R  V  A  G  L
     1060         •       1080        •       1100        •       1120        •
                    •       1140        •       1160        •       1180        •       1200
    1121 TTGAGACCATCTCTACAGCCACAGGGCGGAAACTGCTGGTGTCTGGGTGGTGGGGTATGGTCCGCCATCCCAACTATCTT 1200
         ||||||||||||||||||||||||||||||||||||||||||||||||||||||||||||||||||||||||||||||||
    1139 TTGAGACCATCTCTACAGCCACAGGGCGGAAACTGCTGGTGTCTGGGTGGTGGGGTATGGTCCGCCATCCCAACTATCTT 1218
           E  T  I  S  T  A  T  G  R  K  L  L  V  S  G  W  W  G  M  V  R  H  P  N  Y  L
     1140         •       1160        •       1180        •       1200        •
                    •       1220        •       1240        •       1260        •       1280
    1201 GGAGACCTCATCATGGCTCTGGCTTGGTCCTTGCCCTGCGGGGTGTCACACCTGCTGCCCTACTTCTACCTCCTCTACTT 1280
         ||||||||||||||||||||||||||||||||||||||||||||||||||||||||||||||||||||||||||||||||
    1219 GGAGACCTCATCATGGCTCTGGCTTGGTCCTTGCCCTGCGGGGTGTCACACCTGCTGCCCTACTTCTACCTCCTCTACTT 1298
          G  D  L  I  M  A  L  A  W  S  L  P  C  G  V  S  H  L  L  P  Y  F  Y  L  L  Y  F
     1220         •       1240        •       1260        •       1280        •
                    •       1300        •       1320        •       1340        •       1360
    1281 CACCGCGCTGCTGGTGCACCGTGAGGCCCGGGATGAGCGGCAGTGCCTGCAGAAGTACGGCCTGGCCTGGCAGGAGTACT 1360
         ||||||||||||||||||||||||||||||||||||||||||||||||||||||||||||||||||||||||||||||||
    1299 CACCGCGCTGCTGGTGCACCGTGAGGCCCGGGATGAGCGGCAGTGCCTGCAGAAGTACGGCCTGGCCTGGCAGGAGTACT 1378
           T  A  L  L  V  H  R  E  A  R  D  E  R  Q  C  L  Q  K  Y  G  L  A  W  Q  E  Y  C
     1300         •       1320        •       1340        •       1360        •
                    •       1380        •       1400
    1361 GCCGGCGTGTGCCTTACCGCATCATGCCCTACATCTACTGA                                        1401
         |||||||||||||||||||||||||||||||||||||||||
    1379 GCCGGCGTGTGCCTTACCGCATCATGCCCTACATCTACTGA                                        1419
           R  R  V  P  Y  R  I  M  P  Y  I  Y  *
     1380         •       1400        •

   % Identity =  89.7 (1257/1401)

 ///
```
